# Mitral Stenosis Presenting with Acute Hearing Loss

**DOI:** 10.1371/journal.pmed.0030233

**Published:** 2006-06-27

**Authors:** Chamutal Gur, Gadi Lalazar, Guy Raphaeli, Dan Gilon, Eldad Ben-Chetrit

## Abstract

A 47-year old woman presented with acute hearing loss, followed by dyspnea and then reduced consciousness and a hemiparesis. Investigations led to a diagnosis of cardiac emboli related to rheumatic mitral stenosis.

## Presentation of Case

A 47-year-old woman was hospitalized in the ear, nose, and throat department of another hospital due to acute left ear hearing loss and tinnitus. General physical examination was not performed. However, audiometric testing on admission showed a sensorineural hearing reduction of up to 60 dB at 1,000-Hz frequency on the left side, with no speech discrimination. There were no abnormalities in saccades (see Glossary), pursuit, or nystagmus. Extensive blood tests, including electrolytes, kidney and liver function tests, complete blood count, and coagulation profile, were all normal. Immunologic serology for antinuclear antibodies, rheumatoid factor, C-ANCA, P-ANCA, anticardiolipin antibodies, and lupus anticoagulant were also negative. Head computed tomography (CT) scan with and without contrast medium did not show any pathology. The patient was treated with fluids and aspirin, and a left laser myringotomy was performed to allow topical steroid application. The patient was discharged, and an ambulatory head magnetic resonance imaging (MRI) scan was ordered.

Three days later, a follow-up pure-tone audiogram showed no improvement in her hearing, and treatment with oral dexamethasone (12 mg/day) was initiated. Two days later, she developed progressive shortness of breath, a dry cough, and edema of her face and legs, and presented to our emergency department. The patient denied fever, chest pain, palpitations, or other systemic symptoms. There was no personal or familial history of cardiovascular disease or a hypercoagulable state.

On examination, the patient was tachypneic with normal pulse rate and blood pressure. She had an accentuated first heart sound, an opening snap with a 3/6 rumbling diastolic murmur, inspiratory pulmonary rales at the lung bases, hepatomegaly, and bilateral mild pedal edema. Neurological examination was normal.

Laboratory tests showed mild normocytic anemia (her hemoglobin was 100 g/l [normal range 120–160 g/l]), with normal white cell and platelet count. Arterial blood gases showed hypoxemia (59 mm Hg [normal range 95 ± 5 mm Hg]) with desaturation (91% [normal range 93–100%]) and mild respiratory alkalosis (pH −7.48 [normal range 7.35–7.45]). Electrocardiography showed normal sinus rhythm with a “mitral” p wave in lead II. Chest X-ray showed moderate pulmonary congestion. Diuretic treatment was initiated, and her respiratory condition improved.

**Figure 1:**
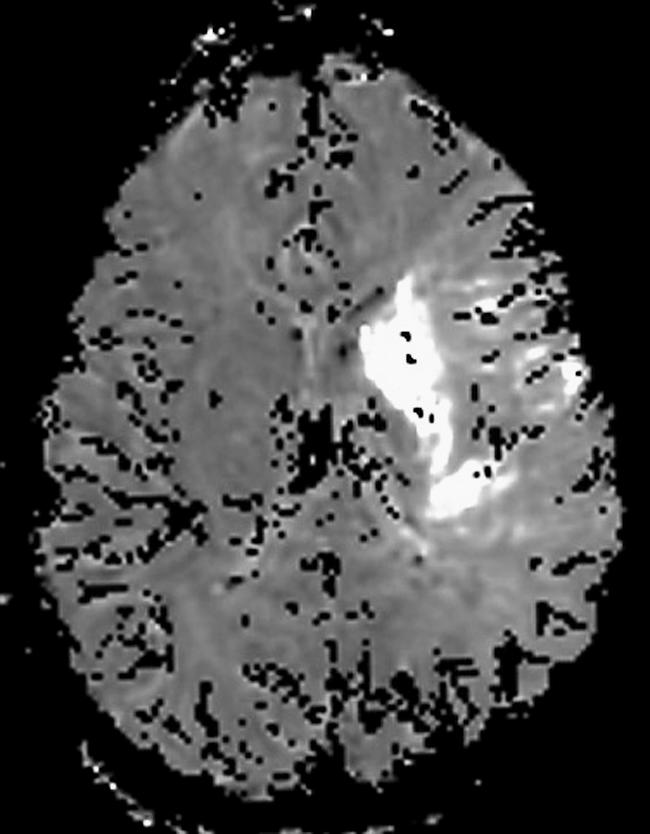
Diffusion-Weighted Imaging (DWI) of the Brain Diffusion defect indicating irreversible left hemispheric ischemic damage in the area controlling right hand and leg function.

**Figure 2:**
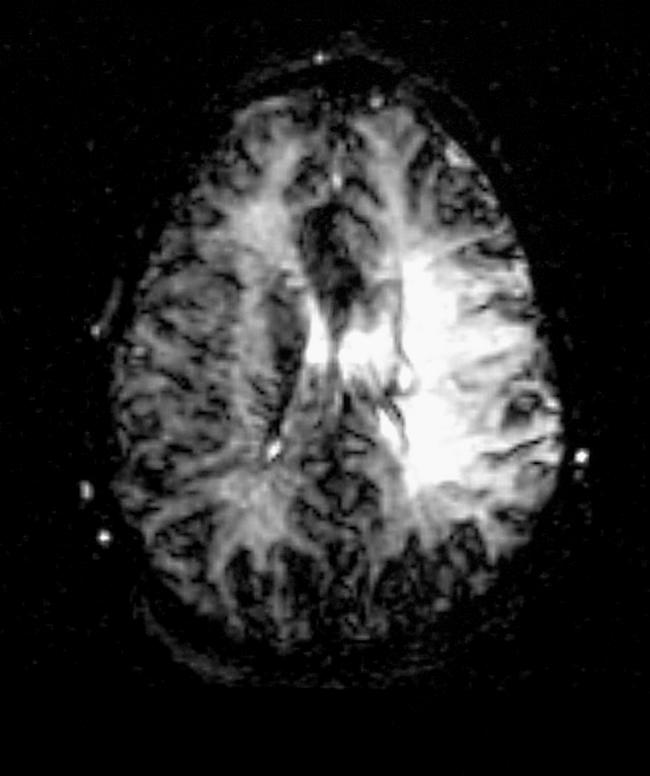
Perfusion-Weighted Imaging (PWI) of the Brain Perfusion amplified signal mismatch (compared to Figure 1), indicating an ischemic area with abnormal function, but no cellular death, salvageable by reperfusion.

**Figure 3:**
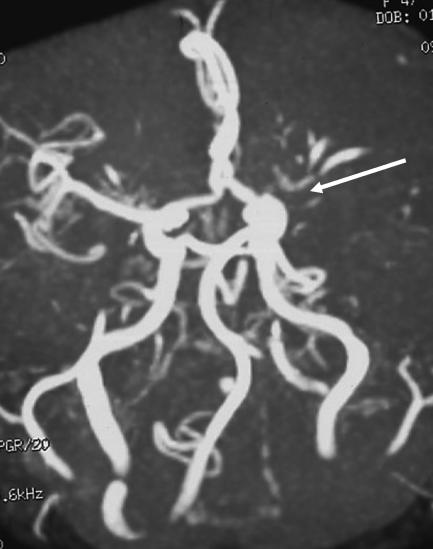
Magnetic Resonance Angiography (MRA) MRA shows the left MCA occluded by a thrombus at its origin (white arrow). This technique uses MR to demonstrate blood vessels within the central nervous system.

**Figure 4:**
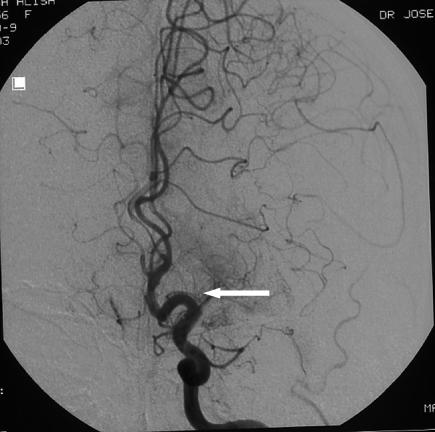
Angiography of the Left Anterior Circulation of the Brain Angiography shows the left internal carotid artery with an occlusion of one of its main branches, the left MCA (arrow shows origin occluded by a thrombus).

**Figure 5:**
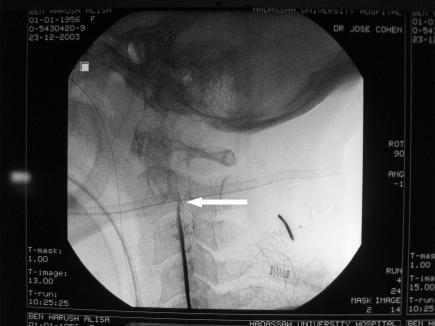
Angiography of the Left Posterior Circulation of the Brain Angiography (sagittal view) demonstrates complete occlusion of the left vertebral artery (black arrow).

The patient was admitted to our department of medicine. Several hours after her admission, she suddenly collapsed. On examination, she had a depressed level of consciousness, global aphasia, left deviating gaze, and right flaccid paralysis with pyramidal signs. The clinical suspicion was of hyper-acute left middle cerebral artery (MCA) occlusion. Electrocardiogram showed normal sinus rhythm. An emergent brain CT scan did not show evidence of infarction or other focal pathology. An emergent MRI protocol including T1-weighted, T2-weighted, and diffusion- and perfusion-weighted imaging (DWI and PWI) was performed, indicating an ischemic area ( Figures 1 and 2). MRA showed a major occlusion of the left MCA at its origin ( Figure 3). Selective angiography showed complete occlusion of the left MCA and left vertebral artery, and incomplete revascularization of the left -MCA territory through leptomeningeal collateral supply (anterior and posterior communicating arteries) ( Figures 4 and 5).

**Figure 6:**
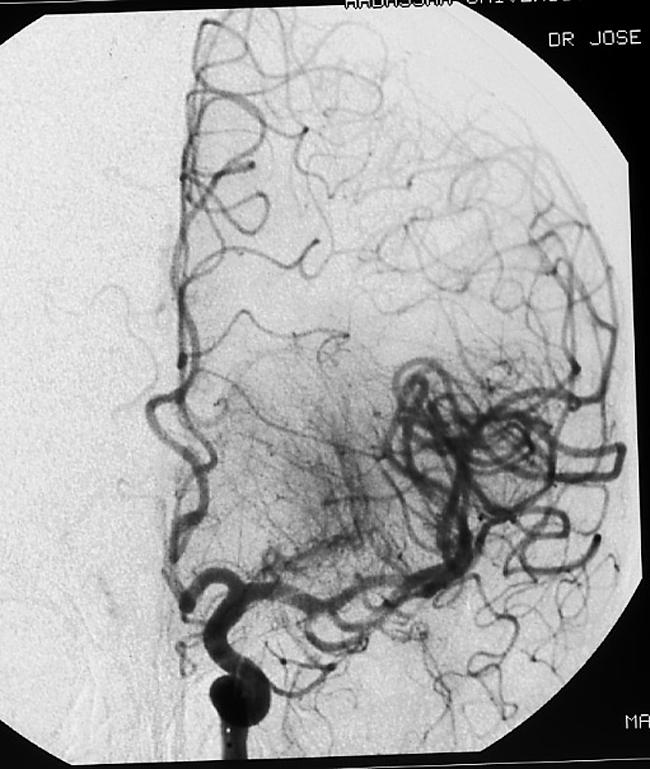
Angiography of the Left Anterior Circulation of the Brain—Following Thrombolysis Antero-posterior view demonstrates a renewed flow in the left internal carotid artery and the left hemisphere.

A 6 French (envoy MPC) 90-cm guiding catheter was placed in the cervical left internal carotid artery. Complete recanalization of the left MCA and its branches was achieved by thrombolysis with urokinase ( Figure 6).

**Figure 7:**
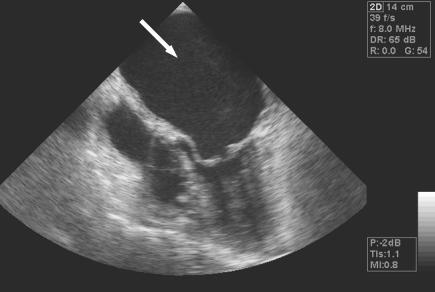
Trans-Esophageal Echocardiogram (TEE) TEE view of significant mitral stenosis (thickening of the mitral valve leaflets with doming of the anterior leaflet) with severe spontaneous echo contrast (white arrow).

Following thrombolysis, the patient regained full consciousness, but continued to have slight right leg weakness and persistent left ear hearing loss. She was treated with heparin followed by warfarin. A trans-esophageal echocardiogram (TEE) performed in the intensive care unit ( Figure 7) showed normal left and right ventricle size and function, moderate mitral stenosis (valve area 1.4 cm ^2^), and mild mitral regurgitation with thickened mitral leaflets. Severe left atrial enlargement (5.1 × 7.0 cm) with moderate spontaneous echo contrast and a small protuberance (2 × 5 mm) on the left atrial side of the inter-atrial septum, compatible with a residual thrombus, were also shown. There was no evidence of vegetations or a patent foramen ovale. 24-hour electrocardiogram monitoring (Holter test) showed normal sinus rhythm (rate 56–117/minute) with occasional atrial premature beats.

The patient was discharged with warfarin treatment and a recommendation for mitral valve replacement. A year later, the patient is asymptomatic and has so far declined mitral valve surgery.

## DISCUSSION

The patient presented with acute hearing loss as a result of cardiac emboli to the posterior circulation (vertebrobasilar territory) and subsequent acute MCA stroke due to emboli to the anterior circulation, both documented by DWI and PWI ( Figure 1). Following investigation into the cause of her multiple emboli, she was diagnosed as having rheumatic mitral stenosis with normal sinus rhythm.

Cardioembolic cerebrovascular events account for 15% to 20% of the 550,000 strokes that occur annually in the United States [ 1, 2]. Strokes due to cardioembolism are generally severe and prone to early recurrence. The emboli typically flow to the intracranial vessels and cause massive, single, large striatocapsular or multiple infarcts in the MCA territory. Decreased consciousness at onset, and simultaneous or sequential strokes in different arterial territories point to a cardiac origin of the stroke [ 2– 4]. This is especially true in cases where both hemispheres or combined anterior and posterior circulations are involved.

Cerebral thromboembolism is a serious complication of mitral stenosis occurring in 13% to 26% of the patients with this lesion [ 5, 6]. In about 12% of these patients, the embolic event is the first presentation of the valvular disease [ 7]. The proposed mechanism is left atrial enlargement, stasis of blood, and increased clot formation in the left atrium and left atrial appendage. The most common risk factors for clot formation are increasing age and the presence of atrial fibrillation [ 8]. Nevertheless, mitral stenosis is associated with embolism to the brain even when the patient is in sinus rhythm.

Spontaneous echo contrast was found to be an important predictor of systemic embolization, independent of the presence of a clot in the left atrium. It is probably associated with evidence of a hypercoagulable state and is believed to represent erythrocyte aggregation (rouleaux formation) in the setting of sluggish blood flow and low shear rate conditions [ 9, 10].

Acute hearing loss (defined as a decrease of more than 20 dB in sensorineural hearing loss occurring over the course of minutes to hours) is a rare symptom of ischemia of the vertebrobasilar system. It is caused primarily by occlusion of the anterior inferior cerebellar arteries or their branches. The auditory system from the cochlea to the central auditory connection in the brain stem is supplied by the vertebrobasilar circulation. There have been several reports of deafness due to vertebrobasilar thromboembolism presenting abruptly and occurring unilaterally. Most of these reports describe associated neurological symptoms, such as a change in mental status and motor weakness, which occur simultaneously [ 11, 12]. However, there are a few case reports [ 13, 14] of acute hearing loss as either the sole manifestation or a warning sign of an impending stroke, as in the case of our patient. To the best of our knowledge, this is the first case report of acute hearing loss as the presenting symptom of mitral stenosis.

**Glossary**

**Saccades:** Small, rapid, jerky movements of the eye, especially as it jumps from fixation on one point to anoter

**Pursuit:** Continuous fluid movement of the eye when tracking an object

**Nystagmus:** Involuntary, usually rapid, movement of the eyes from side to side; nystagmus is a sign of brain injury/disease, although it is also normal for it to occur with dizziness, and during and after bodily rotation.

**Learning Points**

Valvular lesions of the heart can be detected by simple cardiac auscultation.

Had our patient been diagnosed with mitral stenosis during her initial hospitalization in the ear, nose, and throat department, she might have been referred for an echocardiographic examination earlier and for subsequent anticoagulant treatment. Such a course may have prevented the subsequent major stroke.

CT scan failed to detect the pathology on two separate occasions. The immediate use of a DWI–PWI MRI rather than a CT scan should be considered in hyperacute strokes.

Multi-territorial infarcts in general and acute hearing loss in particular can be presenting symptoms of mitral stenosis even in patients with normal sinus rhythm.

Systemic anticoagulation should be considered in patients with significant mitral stenosis even if they are in sinus rhythm.

## Abbreviations

CT: computed tomography
DWI: diffusion-weighted imaging
MCA: middle cerebral artery
MRI: magnetic resonance imaging
PWI: perfusion-weighted imaging
TEE: trans-esophageal echocardiogram
